# Effects of an 8-month intergenerational adapted taekwondo training on functional fitness in senior novice practitioners

**DOI:** 10.3389/fragi.2026.1849400

**Published:** 2026-07-03

**Authors:** Francesca Di Rocco, Andrea Perazzetti, Emanuel Festino, Olga Papale, Simone Ciaccioni, Philip X. Fuchs, Veronica Del Duca, Salvatore Chiodo, Laura Capranica, Cristina Cortis, Andrea Fusco

**Affiliations:** 1 Department of Human Sciences, Society and Health, University of Cassino and Lazio Meridionale, Cassino, Italy; 2 European University of Technology EUt+, Cassino, Italy; 3 Department of Human Sciences and Promotion of Quality of Life, “San Raffaele” Open University of Rome, Rome, Italy; 4 Degree Course in Sciences of Motor Activities, Sports and Psychomotor Education, Faculty of Law, Telematic University Giustino Fortunato, Benevento, Italy; 5 Department of Movement, Human and Health Sciences, University of Rome “Foro Italico”, Rome, Italy; 6 Department of Physical Education and Sports Sciences, National Taiwan Normal University, Taipei, Taiwan; 7 Department of Experimental and Clinical Medicine, University “Magna Græcia” of Catanzaro, Catanzaro, Italy; 8 Italian Taekwondo Federation (FITA), Rome, Italy; 9 Department of Medicine and Aging Sciences, University of “G. D’Annunzio” Chieti-Pescara, Chieti, Italy

**Keywords:** combat sports, elderly, martial arts, older adults, physical activity

## Abstract

**Background:**

Aging is commonly associated with declines in functional fitness that can be effectively attenuated through mid-to long-term, multifaceted physical activity. However, physical activity performed by older adults often aims to maintain, rather than improve, physical fitness. Martial arts are increasingly recognized as suitable multimodal interventions for older adults, as they integrate balance, flexibility, motor control, and cognitive engagement. Within this framework, taekwondo may represent a feasible activity to preserve and enhance functional fitness. Moreover, intergenerational approaches (combining younger and older participants in shared training sessions) may further support adherence, motivation, and social-cognitive stimulation. Despite this potential, long-term programs involving novice older adults remain largely unexplored. Therefore, this study aimed to evaluate the effect of an 8-month intergenerational adapted taekwondo training program for older novice practitioners.

**Methods:**

This single-arm pre–post observational study used a volunteer convenience sample without a control group. Twenty-one seniors (14 females and 7 males: 63–83 years) participated twice-weekly 60-min taekwondo training for 8 months with one weekly session performed together with 21 children (6–13 years). The “American Alliance for Health, Physical Education, Recreation, and Dance” test battery was used to assess the seniors’ functional fitness before and after the intervention based on flexibility, upper-body strength, aerobic endurance, agility/dynamic balance, and coordination. Linear mixed-effects models for repeated measures examined time and sex effects.

**Results:**

Significant (p < 0.05) improvements were observed in aerobic endurance and coordination, with normative compliance increasing from 14.3% to 33.3% and from 66.7% to 95.2%, respectively. No significant changes were found in upper body strength, agility/dynamic, or flexibility, though males outperformed females in the Chair sit-and-reach and endurance tests.

**Discussion:**

Despite the limited sample size and the lack of a control group, which limit causal inference and generalizability, the program was associated with improvements in selected functional fitness domains, particularly aerobic endurance and coordination, whereas flexibility, agility/dynamic balance, and upper-body strength did not change significantly, suggesting that intergenerational adapted taekwondo may represent a feasible long-term physical activity option for novice older adults.

## 1 Introduction

Aging is often accompanied by physiological changes, such as sarcopenia, reduced neuromuscular function, and impaired balance, increasing the risks of falls, injuries, and hospitalization (Duque, 2016; Rodrigues et al., 2022). Regular physical activity supports healthy aging and independent living in older adults (Ahn et al., 2009; Kwak and Cho, 2017), whereas inactivity is linked to chronic diseases, cognitive decline, and lower quality of life (Petrovic, 2017; Melhim, 2001). In accordance with the World Health Organization (WHO) recommendations (Bull et al., 2020), structured exercise programs focusing on functional fitness are considered essential to counteract age-related physical and cognitive decline (Glass et al., 2002; Kim et al., 2022; Ciaccioni et al., 2024c; Antonucci et al., 2014; Baek et al., 2021; St Monica Trust., 2018). Functional fitness is a multidimensional construct, encompassing a set of measurable health- and skill-related components including body composition, cardiorespiratory fitness, muscular strength, flexibility, balance, agility/dynamic balance, coordination, and walking speed (Navarrete-Villanueva et al., 2020). These abilities are crucial for meeting ordinary and unexpected demands of daily life safely and effectively, and lower functional fitness is strongly associated with frailty (Navarrete-Villanueva et al., 2020). Based on principles of reliability, criterion-validity, predictive validity, feasibility, and safety, the “American Alliance for Health, Physical Education, Recreation, and Dance” (AAHPERD) test battery (Osness, 1990) and the Senior Fitness Test (SFT) Battery (Rikli and Jones, 2013) were developed as field-based functional fitness-test batteries and stratified norms by sex and age, which have been extensively used to evaluate the functional fitness level of older individuals and as a benchmark to contextualize changes in functional fitness throughout aging (Cossio-Bolaños et al., 2024). Importantly, functional fitness has a clear practical value in older adults, as higher levels of physical efficiency directly support autonomy in activities of daily living, sustained participation in community life, and overall quality of life in the context of a rapidly aging society (Melhim, 2001; Ahn et al., 2009; Kwak and Cho, 2017; Petrovic, 2017; Navarrete-Villanueva et al., 2020). From a public health perspective, interventions targeting functional fitness may therefore contribute not only to health outcomes, but also to maintaining independence and reducing the risk of disability in later life.

Given the importance of preserving functional fitness in later life, growing attention has been directed toward physical activity interventions able to simultaneously stimulate physical, cognitive, and social domains. Among various forms of health-enhancing physical activity interventions, structured group activities have gained attention for their potential to enhance physical, psychological, and social wellbeing (Ciaccioni et al., 2024c; 2024b). The European Union has also encouraged intergenerational initiatives that involve younger and older individuals to foster mutual understanding, community cohesion, and to help maintain social inclusion, physical fitness, and psychological wellbeing in seniors (European Commission, 2020). Recent literature (Kim et al., 2022; Ciaccioni et al., 2024b; 2024c) has further emphasized the value of intergenerational and martial arts-based approaches for healthy aging, highlighting their potential to support physical function, social engagement, and implementation in older-adult settings. In particular, intergenerational programs proved to foster social interactions and interpersonal relationships (i.e., friendship), reduce loneliness and depression, improve attitudes towards young people, and promote motivation in older individuals (Canton-Martínez et al., 2024; Loyola et al., 2018; Maugeri et al., 2020; Buonsenso et al., 2021). By involving complex and dynamic movements that can enhance balance, coordination, and muscular strength, martial arts have been shown to enhance psychological wellbeing, functional fitness, and postural stability in aging populations, which may support a holistic approach to physical and mental health and overall wellbeing (Ciaccioni et al., 2024c; Li et al., 2024). Furthermore, in intergenerational settings, martial arts practice combines benefits for functional fitness and wellbeing by offering complex, multi-faceted motor challenges fostering motivation, social cohesion, and interaction between generations (Piercy et al., 2018; Bull et al., 2020; Stamatakis and Bull, 2020).

Considering the increasing evidence supporting martial arts as an effective tool for healthy aging, the evaluation of their application in intergenerational contexts could be of interest. Review-literature suggests that intergenerational physical activity may benefit older adults not only through physical engagement, but also by fostering social interaction, motivation, cognitive stimulation, and psychological wellbeing (Krzeczkowska et al., 2021; Kim et al., 2022; Ciaccioni et al., 2024c). In parallel, reviews on martial arts-based interventions in middle-aged and older populations indicate that these practices may support functional fitness, balance, coordination, mobility, and general wellbeing when appropriately adapted to participants’ characteristics and safety needs (Origua Rios et al., 2018; Palumbo et al., 2023; Ciaccioni et al., 2024c). Adapted intergenerational taekwondo may thus represent a meaningful multimodal activity for novice older adults because it combines repeated whole-body motor tasks with social and relational engagement. Specifically, stance work, weight transfer, upper- and lower-limb techniques, and movements performed through large ranges of motion may provide stimuli relevant to physical development and functional fitness, whereas shared practice with younger partners may enhance adherence, attention, enjoyment, and mental engagement. Although the present study did not directly assess mental fitness or psychosocial outcomes, these dimensions contributed to the theoretical rationale for considering intergenerational adapted taekwondo as a potentially relevant activity for older populations. Therefore, this single-arm pre-post observational study aimed to assess the effects of an 8-month twice-weekly intergenerational adapted taekwondo training program on functional fitness in senior novice practitioners using field-based functional fitness tests. It was hypothesized that the intergenerational taekwondo intervention would be associated with improvements in key functional fitness domains, such as strength, flexibility, and coordination, in both female and male community-dwelling seniors.

## 2 Materials and methods

### 2.1 Participants

The study was conducted in accordance with the Declaration of Helsinki and approved by the Ethics Committee of the University of Rome “Foro Italico” (CAR 144/2023). All participants were volunteers and informed of their right to withdraw from the study at any time without any consequences and asked to sign a written consent form. As required by the Italian Regulation (D.M. 28/2/1983), participants provided a medical certification for engaging in physical activity. In accordance with the European Parliament Regulation (EU 2016/679) on General Data Protection Regulation (GDPR), personal data were anonymized by assigning a unique identification code, with data solely used for statistical purposes. Inclusion criteria encompassed: community dwelling individuals older than 60 years of age with no previous experience in taekwondo, no cardiovascular, respiratory, and metabolic diseases, and/or musculoskeletal injuries of the back or lower extremities.

A volunteer convenience sampling approach was used in this study. Community-dwelling older adults from the local area were recruited through the collaborating training center A.S.D. Taekwondo Hwarang-Silla (Terracina, Italy) and its community network as part of the Italian Taekwondo Federation (FITA) affiliated initiative. Interested individuals were screened for eligibility according to the predefined inclusion criteria and the required medical clearance for physical activity, and all eligible volunteers who provided written informed consent were enrolled. Each senior participated together with a grandchild or another relative child, forming intergenerational pairs.

A total of 21 seniors (aged: 63–83 years) and 21 of their grandchildren or young relatives (aged: 6–13 years) were recruited, with one child for every senior. All children held a yellow belt rank (i.e., second belt level in the color taekwondo skill progression system), indicating a beginner level of taekwondo proficiency. The STROBE reporting guidelines for observational studies were used (Vandenbroucke et al., 2014).

### 2.2 Procedures

Participants completed 60-min taekwondo training sessions twice a week for 8 months at the training center A.S.D. Taekwondo Hwarang-Silla (Terracina, Italy), organized according to the National program to support post COVID-19 pandemic physical active lifestyle (FITA, Sport e Salute S.p.A. CDR2.PNR2023_DM737/2021TA). Older participants were exempt from the registration fee and medical check payment. Furthermore, they were provided with a Taekwondo kit (Dobok), whereas youth participants received a 20% discount on the registration fee as part of the project support. No financial incentives were provided to older participants. The taekwondo training program was in line with the most recent international physical exercise recommendations for older adults (Bull et al., 2020).

Each training session comprised three phases: a 10-min warm-up, a 40-min central phase and a 10-min cool down. The warm-up routines encompassed walking at different speeds and/or performing taekwondo arm techniques while standing, aimed to prevent exercise-induced musculoskeletal injuries and cardiac events in older individuals (Buckley et al., 2008; Mazzeo and Tanaka, 2012; DelCastillo-Andrés et al., 2018). Given the novice status of the older participants, the initial phase of the program also served as an orientation period to the exercise environment and to ensure the safe execution of the basic motor patterns required during training. The central phase was divided into two sections and encompassed: (i) general conditioning targeting balance and strength, and (ii) taekwondo-specific technique. The conditioning section lasted approximately 25 min and consisted of a circuit of 10 exercises performed in 3 series. Representative exercises included squats, single-leg balance tasks, and front raises using a belt. Across the intervention, this component was conservatively progressed by increasing work intervals from 30 to 50 s and reducing inter-series recovery from 2 min to 1 min and 30 s, while maintaining a low-to-moderate intended intensity appropriate for novice older adults. Progression was implemented following the principles of safety, progression and proficiency (e.g., from slow to fast, simple to complex, known to unknown, low to moderate intensity, static to dynamic) (Skelton, 2006; Rikli and Jones, 2013). Exercise volume and technical complexity were adapted by the instructors according to individual tolerance and motor competence, rather than being prescribed as a fixed technical number of repetitions for all participants. The 10-min cool down phase included low-intensity taekwondo techniques, and upper and lower body stretching while focusing on calm and slow breathing to allow for gradual and safe recovery and prevent dizziness and cardiorespiratory irregularities (Buckley et al., 2008).

Safety was ensured through continuous supervision by two certified FITA instructors (V.D.D. and S.D.L.) and three PhD researchers (F.D.R., E.F., and O.P.), together with conservative progression tailored to older novice participants. This supervision structure allowed continuous monitoring of both children and older participants during the sessions, and prompt technical corrections. Furthermore, participants were routinely verbally reminded during the training sessions to report any musculoskeletal discomfort, dizziness, or unusual cardiorespiratory symptoms. In such cases, the activity was immediately stopped or modified. Before starting the intervention, to ensure correct understanding, older participants received standardized instructions and were familiarized with the Italian version of the Borg category-ratio 10 scale (CR-10) (Arney et al., 2019) including its verbal anchors (0 = rest/no exertion, 10 = maximal exertion). During an initial familiarization session, participants practiced providing a global rating of the overall session intensity. To monitor internal training load, 30 min after each session the participants reported their perceived intensity of the session, and the values were multiplied by the duration of the session in minutes (Foster et al., 2021) to provide the session Rating of Perceived Exertion (sRPE). Hence, sRPE values were used to confirm that sessions were delivered within the intended intensity range across the intervention period.

One weekly session was administered only to the seniors and focused on balance, strength, and sport-specific skill development involving taekwondo punches (e.g., jumok jirugi), kicks (e.g., Ap-chagi, Jiko-chagi), blocks (e.g., Bakat palmok are makki, Palmok momdong an makki), and stances (e.g., Ap-sogi, Ap-kubi). The second weekly session also included the children and focused on the interaction and collaboration between younger and old participants by practicing exercises in intergenerational pairs, with one practitioner executing a specific technique, and the other partner reacting accordingly. For example, children performed an ap-chagi, whereas seniors stepped back and executed bakat palmok are makki. This approach is common in taekwondo training to improve cooperation, memory capacity, and reaction between partners. A structured overview of the intervention content and progression is provided in Table 1.

**TABLE 1:** Structured overview of the 8-month intergenerational adapted taekwondo intervention.

Session component	Content/representative exercises	Progression/implementation
Warm-up (10 min)	• Walking or stepping tasks • Walking in place • Simple taekwondo arm techniques	Used to prepare participants for the session. During the initial phase of the program, this component also served as orientation to the exercise setting and to safe movement execution
Conditioning section, approximately 25 min within the 40-min central phase	Circuit of 10 exercises targeting balance and strength Representative exercises included squats, single-leg balance tasks, and front raises using a belt	Performed in 3 series. Work intervals were progressively increased from 30 to 50 s, and inter-series recovery was progressively reduced from 2 min to 1 min and 30 s. Intended intensity was maintained at a low-to-moderate level
Senior-only technical practice	Basic taekwondo techniques, including: 1. Blocks (makki): • High block with external forearm (bakat palmok olgul makki) • Medium block with internal forearm (palmok momdong an makki) • Medium block with external forearm (bakat palmok momdong makki) • Lower block with external forearm (bakat palmok are makki) 2. Predefined sequences of movements stimulating responses to directional stimuli (First Poomsae) 3. Stances (sogi): • Legs about 40 cm apart with arms relaxed at the sides (cyariot-sogi) • Standing with one foot about 30 cm forward (ap-sogi) • One foot approximately 70 cm forward with the front knee flexed (ap-kubi) • combat stance, with a foot about 40 cm forward, bent knees, and bent arms at chest height (kyeorumse) 4. Kicks (chagi): • front kick (ap-chagi) • descendant kick (jiko-chagi) • push kick (miro-chagi)	Emphasis placed on technical acquisition, safe execution, and conservative progression from simple to more integrated motor tasks
Intergenerational technical and collaborative practice	• Music-based circle activities • Cooperative circle activities • Paired block-kick combinations • Balance and strength tasks performed in pairs • Kicking on pads • Occasional pair-based games using taekwondo techniques	Emphasis placed on interaction, cooperation, reaction, and collaborative engagement between children and older participants
Cool-down (10 min)	Slow walking and static stretching for quadriceps, calf muscles, shoulders, and ankles	Used to promote gradual recovery and safe return to resting conditions
Individualization	All session components adapted according to individual tolerance, motor competence, and instructor judgment	No identical fixed number of repetitions prescribed for all participants

Progression was conservative and tailored to novice older adults to prioritize safety, adherence, and technical proficiency.

**FIGURE 1:**
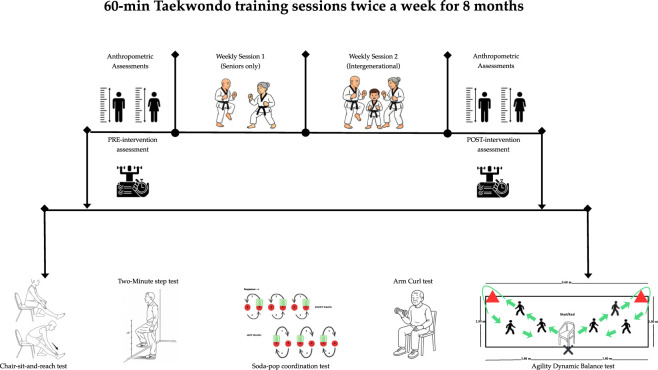
Schematic overview of the study design and procedures of the 8-month intergenerational adapted taekwondo training program, including PRE- and POST-intervention anthropometric assessments and the functional fitness tests administered.

To collect anthropometric and fitness data, the senior participants attended two measurement sessions administered at the beginning (PRE) and at the end (POST) of the intervention. Before each measurement session, the participants were familiarized with the experimental procedure (Figure 1).

### 2.3 Anthropometric measurements

The investigators followed the international standards for anthropometric assessment (Marfell-Jones et al., 2006) by collecting height (cm) and body mass (kg) of each participant in underwear and barefoot standing on a scale with an integrated stadiometer (Seca, model 709; Vogel & Halke, Hamburg, Germany) with an accuracy of ± 0.1 kg for body mass and ± 0.1 cm for height. Body mass index (BMI) was calculated as weight divided by height squared (kg·m^−2^). Anthropometric measurements were collected as widely used indicators to describe participants’ weight status in this field-based community intervention, while minimizing participant burden and maximizing feasibility over the 8-month program. Table 2 summarizes participant anthropometric characteristics.

**TABLE 2:** Mean values and standard deviations of participant characteristics before (PRE) and after (POST) the 8-month intergenerational taekwondo training program.

Variables	Females (*n* = 14)		Males (*n* = 7)	
​	PRE	POST	PRE	POST
Age (years)	72.4 ± 6.5	72.4 ± 6.5	75.1 ± 3.8	75.1 ± 3.8
Body height (cm)	154.3 ± 7.7	154.3 ± 7.7	165.5 ± 6.8	165.5 ± 6.8
Body mass (kg)	71.3 ± 12.9	71.9 ± 13.8	68.7 ± 7.5	68.3 ± 6.4
BMI (kg·m^-2^)	30.0 ± 5.4	30.3 ± 6.0	25.1 ± 2.7	24.9 ± 1.8

n, number; BMI, body mass index.

### 2.4 Functional fitness assessment

The functional fitness of participants was assessed using a combination of tests derived from two validated and widely used batteries. Specifically, tests of upper body strength (i.e., Arm curl), agility and dynamic balance (i.e., agility/dynamic balance), and manual dexterity (i.e., Soda-pop coordination) were selected from the AAHPERD test battery (Osness, 1990), whereas tests of lower body flexibility (i.e., Chair sit-and-reach) and aerobic endurance (i.e., Two-minute step) were selected from the Senior Fitness Test (Jones and Rikli, 2002). All tests were administered according to the standardized protocols detailed in the original sources (Osness, 1990; Jones and Rikli, 2002), and the assessments were performed in a randomized order. Tests were not combined into a composite score nor used for between-battery comparisons. Each outcome was analyzed in its original unit and interpreted within specific domains. The tests administered are described below.

- Chair sit-and-reach: participants performed 3 trials with 1-min resting in between, with the highest value (cm) considered the best trial and recorded for the analysis.
- Arm curl: participants performed one trial, and the total number (n) of repetitions in 30 s was recorded.
- Agility/dynamic balance: two trials were carried out with 30 s of rest in between, with the lowest value (s) considered the best performance and recorded for the analysis.
- Two-minute step in place: the total number (n) of valid steps (right knee reaching the mid-thigh mark) was recorded.
- Soda-pop coordination: the lower score (s) from two trials was recorded as the best performance for the analysis.

### 2.5 Statistical analysis

Statistical analysis was conducted using the statistical software STATA version 18.0 (Stata-Corp, LLC, College Station, TX, USA). For all variables, means, standard deviations (SD), 95% confidence intervals (95% CI), and changes in normative compliance (Δ) from PRE to POST were calculated. After checking the normal distribution of the data via the Shapiro-Wilk test, linear repeated measures mixed models examined the effects of the 8-month taekwondo intervention on seniors’ functional fitness accounting for sex. The models considered participants as a random effect to account for inter-individual variability, while sex (female, male) and testing time (PRE, POST) were treated as fixed effects. To account for the small sample size, restricted maximum likelihood estimation was used to fit the models. Repeated measures analysis of variance (ANOVA) computed the degree of freedom of a t-distribution since participants were tested twice. The contrast method, ANOVA-style tests of main effects, was subsequently employed to test whether the means of the dependent variables (e.g., arm curl repetitions) were equivalent for sex (female, male) and testing times (PRE, POST). Statistical significance was set at p < 0.05. Bonferroni correction (adjusted p < 0.006) was applied only to *post hoc* pairwise comparisons following significant main or interaction effects. Standard errors (SE) and intraclass correlation coefficients (ICCs) expressed the estimation error of the linear repeated measures mixed models. Effect sizes were calculated and reported as partial eta squared (ηp^2^) for ANOVA effect and interpreted as small (ηp^2^ < 0.06), medium (0.06 ≤ ηp^2^ < 0.14), or large (ηp^2^ ≥ 0.14) (Hopkins et al., 2009).

## 3 Results

The subjective perception of effort revealed a mean RPE of 2.8 ± 0.5, corresponding to low-to-moderate intensity (Borg et al., 1987). The mean sRPE load was 201.7 ± 68.1 AU, confirming that the adapted taekwondo training was perceived as low-to-moderate in terms of exertion by the senior participants.

**FIGURE 2:**
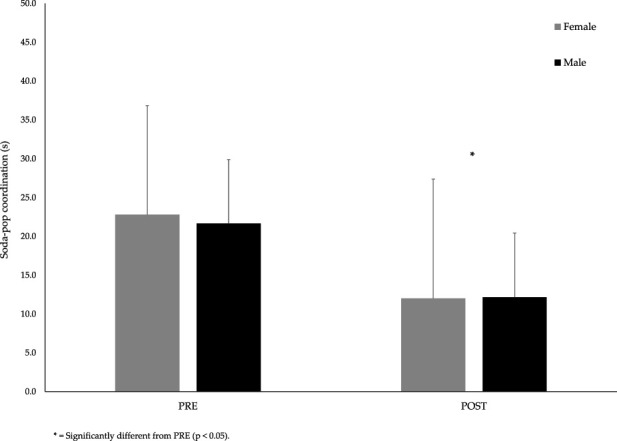
Means and standard deviations of the soda-pop coordination test performance (time, s) for females and males before (PRE) and after (POST) the 8-month intergenerational taekwondo training program. * = significantly different from PRE (p < 0.05).

A significant interaction between sex and time was found for the Chair sit-and-reach test (F_(3,19)_ = 3.03, p = 0.014, 95% CI = 2.06 to 16.00, SE = 3.32, ICC = 0.68). No overall improvement over time was observed (p = 0.455). Post hoc comparisons did not reveal statistically significant differences from PRE to POST in either females (Δ = + 9.04 cm, SE = 3.33, p = 0.040), or males (Δ = −2.71 cm, SE = 2.15, p = 1.000), although females showed a trend toward improved reach distance. Time spent during the Soda-pop coordination test decreased from PRE to POST across the sample (F_(3,19)_ = 16.2, p < 0.0001, 95% CI = −14.60 to −6.96, SE = 1.48, ICC = 0.17), without sex (p = 0.652) and interaction effects (p = 0.689). These changes are reported in Figure 2.

**FIGURE 3:**
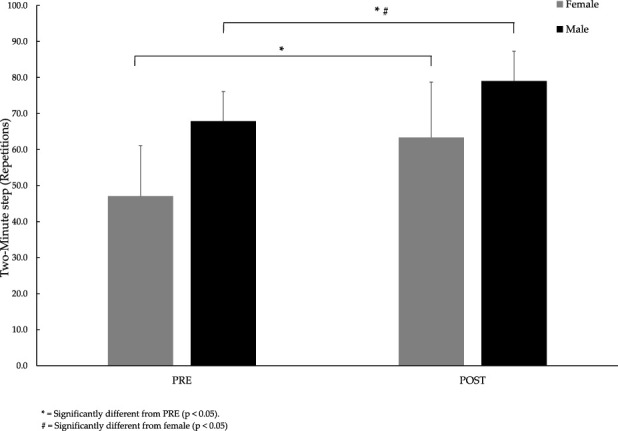
Means and standard devistions of the two-minute step test performance (repetitions) for females and males before (PRE) and after (POST) the 8-month intergenerational taekwondo training program. * = significantly different from PRE (p < 0.05); # = significantly different from female (p < 0.05).

The Two-minute step test score (Figure 3) was higher in males with respect to their female counterparts (F_(3,19)_ = 13.07, p = 0.003, 95% CI = 8.17 to 33.39, SE = 6.02) and in POST than in PRE (p < 0.0001, 95% CI = 9.00 to 23.56, SE = 3.48, ICC = 0.50), with no interaction effect (p = 0.432).

The agility/dynamic balance test (Table 3) showed no main effects for sex (p = 0.449) and time (p = 0.775), and no interaction between sex and time (p = 0.821). The Arm curl test showed no main effect of sex (p = 0.377), time (p = 0.100), and no interaction between sex and time (p = 0.479).

**TABLE 3:** Means and standard deviations of the functional fitness tests performances before (PRE) and after (POST) the 8-month adapted taekwondo training.

​	PRE			POST		
Test	Females (*n* = 14)	Males (*n* = 7)	Total (*n* = 21)	Females (*n* = 14)	Males (*n* = 7)	Total (*n* = 21)
Chair sit-and-reach (cm)	1.4 ± 2.9	10.4 ± 9.8	4.4 ± 7.3	4.1 ± 6.6	7.7 ± 11.0	5.3 ± 8.2
Soda-pop coordination (s)	22.8 ± 8.1	21.7 ± 3.9	22.4 ± 6.9	12.0 ± 2.6*	12.2 ± 2.5*	12.1 ± 2.5
Two-minute step (repetitions)	47.1 ± 14.0	**67.9 ± 8.2#**	54.0 ± 15.8	63.4 ± 15.4*	79.0 ± 8.2*#	68.6 ± 15.2
Arm curl (repetitions)	12.8 ± 3.0	14.7 ± 4.1	13.4 ± 3.4	10.7 ± 5.8	11.1 ± 5.0	10.9 ± 5.4
Agility/dynamic balance (s)	42.6 ± 8.6	39.4 ± 4.5	41.6 ± 7.5	43.1 ± 11.1	40.6 ± 7.8	42.3 ± 10.0

n = number; * = Significantly different from PRE (p < 0.05). # = Significantly different from females (p < 0.05). Statistically significant values are shown in bold.

The proportion of participants meeting sex-specific normative standards at PRE and POST, together with the relative change in normative compliance are reported in Table 4. Normative compliance increased substantially for the Soda-pop coordination test (66.7%–95.2%) and for the Two-minute step test (14.3%–33.3%). In contrast, normative compliance decreased for Chair sit-and-reach test in females (85.7%–57.1%) and for Arm curl test in females and in the total sample (71.4%–47.6%).

**TABLE 4:** Sex-specific normative compliance before (PRE) and after (POST) the 8-month adapted taekwondo training. Values are reported as n (%), and Δ% indicates the relative change in normative compliance from PRE to POST.

​	Females			Males			Total		
Test	PRE *n (%)*	POST *n (%)*	Δ%	PRE *n (%)*	POST *n (%)*	Δ%	PRE *n (%)*	POST *n (%)*	Δ%
Chair sit-and-reach (cm)	12 (85.7)	8 (57.1)	−33.4	2 (28.6)	3 (42.9)	+50.0	14 (66.7)	11 (52.4)	−21.4
Soda-pop coordination (s)	8 (57.1)	13 (92.9)	+62.7	6 (85.7)	7 (100.0)	+16.7	14 (66.7)	20 (95.2)	+42.7
Two-minute step (repetitions)	0 (0.0)	3 (21.4)	—	3 (42.9)	4 (57.1)	+33.1	3 (14.3)	7 (33.3)	+132.9
Arm curl (repetitions)	11 (78.6)	6 (42.9)	−45.4	4 (57.1)	4 (57.1)	0.0	15 (71.4)	10 (47.6)	−33.3
Agility/dynamic balance (s)	2 (14.3)	4 (28.6)	+100.0	1 (14.3)	2 (28.6)	+100.0	3 (14.3)	6 (28.6)	+100.0

n = number; PRE = before intervention; POST = after intervention; Δ% = relative change in normative compliance, computed as ((POST% - PRE%)/PRE%) × 100. Δ% was not computed when PRE% = 0.

## 4 Discussion

This study aimed to evaluate the effects of an 8-month intergenerational taekwondo training program on functional fitness in novice senior practitioners. The main findings highlighted that: 1) significant pre–post improvements were observed in coordination and aerobic endurance; 2) no significant changes were observed in upper body strength, agility/dynamic balance, and lower body flexibility; and 3) males showed higher aerobic endurance but lower gains in flexibility compared to their female counterparts. The lack of significant improvements in flexibility, agility/dynamic balance, and upper-body strength, and the observed decreases in normative compliance in some domains (e.g., flexibility and upper-body strength), should be considered a limitation of the intervention, indicating that this adapted taekwondo program may not provide a sufficient stimulus for these capacities unless complemented with targeted flexibility and resistance-oriented exercises. These results are partially in line with previous studies. For example, Cho and Roh (Cho and Roh, 2019) reported significant improvements in flexibility (i.e., Chair sit-and-reach), aerobic endurance (i.e., Two-Minute step), and upper body strength (i.e., Arm curl) after 16 weeks of taekwondo in older women, whereas the participants of the present study showed improvements in coordination and aerobic endurance only. More recent evidence (Lia et al., 2025) supports the potential of adapted taekwondo to improve functional outcomes in older adults. For example, a 6-month adapted taekwondo intervention was associated with improved functional mobility in healthy older adults, although different outcome measures were compared. Differences between studies may reflect protocol-related factors (e.g., training frequency, intensity, and exercise content), as well as sample characteristics (women-only vs. mixed-sex sample) and baseline functional status. In particular, these differences may depend on the overall training exposure (intervention length, weekly frequency, and session intensity) and on the specificity of the training stimuli. Although the present program lasted 8 months, sessions were delivered at a moderate internal load (mean RPE ≈2.8) and followed a conservative progression, which may have provided a more consistent stimulus for aerobic endurance and coordination than for strength or flexibility adaptations. Improvements in upper-body strength and flexibility typically require higher mechanical loading and/or dedicated resistance- and stretching-focused components, which were not systematically emphasized within the training sessions of the current intervention. The participants of this study showed baseline levels of functional fitness that were on average within the normative ranges reported for older adults (Osness, 1990; Capranica et al., 2001). Despite the relative older age of several participants, the proportion reaching normative thresholds increased in coordination and endurance, suggesting meaningful functional benefits.

Flexibility is crucial for seniors in daily life activities (e.g., walking, reaching, lifting, bending) and it contributes to the prevention and management of low-back pain, musculoskeletal injuries, and gait impairments, to maintaining mobility, balance, and overall physical function, to reduced risks of falls, and to an independent lifestyle (Rikli and Jones, 2013; Seco et al., 2013; Chodzko-Zajko et al., 2009; Currier et al., 2026). However, flexibility progressively deteriorates throughout adulthood, and its preservation typically requires targeted stimuli, such as regular stretching and mobility exercises, which promote joint range of motion and muscle elasticity and may support healthy aging (Ciaccioni et al., 2019). In the present study, females showed a greater PRE-POST improvement than males (Δ = +9.04 cm vs. −2.71 cm). This finding contrasts with the initial hypothesis, although it is consistent with normative references data showing higher flexibility in older women than men (Rikli and Jones, 2013). Given the lack of a significant overall group effect, this sex difference should be interpreted cautiously since it may suggest that taekwondo practice may benefit flexibility more in women, possibly due to sex-specific differences in connective tissue compliance and muscle stiffness (Cho and Roh, 2019; Di Rocco et al., 2023).

Although mean endurance and coordination scores were already within normative ranges at baseline for both females and males, further improvements were observed following the 8-month taekwondo training program. Specifically, the Soda-pop coordination test showed the largest functional gain with most participants reaching normative values after the intervention. The Two-minute step test also demonstrated a meaningful increase in normative compliance, indicating enhanced cardiorespiratory capacity in both females and males. These changes reflect significant individual-level gains in functional fitness, particularly in coordination and aerobic endurance. Previous studies have shown that physical activity, particularly when involving rhythmic, repetitive movements such as taekwondo techniques, can enhance aerobic capacity in seniors (Spirduso and Francis, 2005). Taekwondo incorporates dynamic, full-body movements that engage both the cardiovascular and muscular systems. The training structure, featuring repetitive technical drills, footwork patterns, and controlled movements performed at varying intensities, likely contributed to these improvements. Similar to aerobic exercise programs, repeated taekwondo practice may have provided sufficient aerobic stimulus to improve performance in the Two-minute step test, although physiological mechanisms were not directly assessed. The substantial improvements aligned with previous literature, indicating that structured martial arts training can enhance aerobic capacity and motor coordination through complex movement patterns and cognitive engagement (Ciaccioni et al., 2019). Given the association between declining coordination, manual dexterity, and increased fall risk in seniors (Capranica et al., 2001), these findings suggest the adapted taekwondo may be a promising activity for supporting selected motor domains in older adults. However, future research should examine whether these improvements translate into better performance in daily life activities (e.g., grasping objects, tying shoes) crucial for maintaining independence during lifespan (Di Rocco et al., 2025).

The lack of significant changes in agility/dynamic balance and upper-body strength suggests that the study duration may have been insufficient to obtain measurable gains. This may be attributed to the progressive nature of the taekwondo training program, in which initial sessions prioritized technique acquisition and safety over high-intensity drills. Improving agility typically requires targeted training with specific agility drills (Young et al., 2001), which were not extensively incorporated during these early stages, likely contributing to the lack of measurable improvements. Because the program emphasized technical learning and safe progression, the agility stimulus may not have been sufficiently specific to elicit measurable changes. Although the intervention incorporated multi-directional movements that challenged balance and stability, it may not have been specific enough to enhance agility performance. Future studies should incorporate agility-specific drills and progressions to test whether greater stimulus specificity yields improvements in agility outcomes in novice older adults. Similarly, the Arm curl test results suggested that taekwondo training did not affect upper-body muscular strength, probably due to the training’s primary emphasis on lower-body movements, with insufficient upper-body load provided by arm strike techniques to elicit strength adaptations. Given the typical age-related decline in muscular strength associated with aging, the present findings may also be interpreted as maintenance of upper body functional strength performance over the 8-month period. Previous studies have shown that social support and interactive training environments can enhance adherence and promote higher exercise intensities (Satariano and McAuley, 2003; Huang et al., 2022). The intergenerational nature of the intervention, involving younger practice partners, may have motivated senior participants, leading to greater engagement and effort during sessions. To investigate the underlying motivational mechanisms, future studies could adopt a mixed-methods design that integrates quantitative measures of engagement and effort, with qualitative insights obtained through interviews or focus groups exploring older participants’ perceived sources of motivation and the interpersonal dynamics established with their younger partners (Ciaccioni et al., 2024a; 2025).

Since a control group was not feasible over an 8-month period, norm-referenced values from validated functional fitness test batteries were used to contextualize the observed results (Rikli and Jones, 2013). The proportion of participants falling within normative ranges was examined for each test to complement the analysis of mean scores. At baseline, 66.7% of participants met normative standards in lower-body flexibility, decreasing to 52.4% after the intervention. In the upper-body strength test, normative compliance declined from 71.4% at PRE to 47.6% at POST. This reduction suggests that the adapted taekwondo program did not provide a sufficient stimulus to maintain normative levels of flexibility and upper-body strength in all participants, consistent with the lack of significant mean-level improvements in these domains. Rather than reflecting a paradoxical worsening, these patterns likely indicate that the training load and content were not specifically targeted enough to preserve these fitness components in a portion of the sample, reinforcing the need to complement future programs with dedicated stretching and resistance-oriented exercises. In contrast, significant improvements were observed in coordination and endurance. In the Soda-pop coordination test, normative compliance increased from 66.7% to 95.2%, and in the Two-minute step test from 14.3% to 33.3%. These results are particularly relevant when compared to studies reporting age-related functional decline over similar time periods in sedentary older adults (Capranica et al., 2001). The findings suggest that consistent, structured practice of martial arts-based exercises, especially in an intergenerational setting, may be associated with maintenance or enhancement of selected functional domains in aging populations. Nevertheless, the reduced normative compliance observed for flexibility and upper-body strength in part of the sample should not be interpreted neutrally, as these domains are clinically relevant for daily functioning and fall-risk reduction. These findings suggest that a taekwondo-only stimulus may be insufficient for some fitness components in novice older adults and supports integrating dedicated flexibility and upper-body resistance exercises within future programs. This interpretation is also consistent with recent review-level evidence suggesting that martial arts-based interventions, including adapted taekwondo, may improve functional mobility in older adults, although the overall certainty of evidence remains limited because of small sample and methodological heterogeneity (De Mendonça et al., 2025).

## 5 Limitations

Several limitations should be acknowledged. First, the study used a small volunteer convenience sample, which limits statistical power and generalizability. Therefore, the findings should be interpreted as preliminary and hypothesis-generating. Second, the lack of a control group represents a major limitation, as it prevents causal attribution of the observed changes exclusively to the taekwondo intervention. Although validated normative values were used as a reference framework to contextualize the findings, they cannot replace a control group or strengthen causal inference. Third, the intergenerational family-based pairing may have introduced heterogeneity and selection bias as participants may have had higher motivation or social support than the broader population of community-dwelling older adults. Although the intervention lasted 8 months and included PRE and POST assessments, the study should be interpreted as a single-arm pre-post observational study rather than as a longitudinal design in a stronger methodological sense. The twice-weekly training frequency was selected for pragmatic, safety-related, and feasibility reasons within a community-based adapted program for novice older adults. However, the study was not designed to compare different weekly training frequencies, and no conclusion can be drawn regarding the optimal training dose. Attrition was 0%, as all 21 enrolled older participants completed both PRE and POST assessments. Nevertheless, session-by-session attendance was not systematically recorded, which limits the evaluation of actual training exposure, feasibility, and possible dose-response relationships. Furthermore, anthropometric assessment was limited to height, body mass, and BMI. Therefore, changes in body composition (e.g., fat and lean mass) could not be examined. The study also focused on functional fitness outcomes and did not directly assess functional independence, healthy aging, mental fitness, motivation, satisfaction, or other psychosocial outcomes. Consequently, interpretations regarding these broader constructs should be considered theoretical rather than direct findings. Future studies should integrate psychosocial measures into the study design, ideally through mixed-methods approaches or multidimensional monitoring frameworks, to better understand how relational and motivational factors shape both participation and functional outcomes (Kim et al., 2022). Furthermore, future research should also explore the long-term sustainability of the observed benefits and assess the effects of more strongly emphasized flexibility, strength, and agility components in martial arts-based interventions.

## 6 Conclusion

The present 8-month intergenerational adapted taekwondo training program was associated with improvements in aerobic endurance and coordination in novice older adults, whereas lower-body flexibility, agility/dynamic balance, and upper-body strength did not significantly change. These findings suggest that structured martial arts training may represent a feasible long-term activity option for aging populations. However, given the small sample size, the lack of a control group, volunteer convenience sampling, and participant heterogeneity, the findings should be interpreted with caution. Larger randomized controlled trials are needed to confirm these results and to determine whether more targeted flexibility-, agility-, dynamic balance-, and upper-body strength-oriented components can broaden the functional benefits of this type of intervention.

## Data Availability

The datasets presented in this study can be found in online repositories. The names of the repository/repositories and accession number(s) can be found below: https://github.com/ccortis/DataTKW.git.
